# Supplementary material for: Evaluation of DNA Methylation Profiles of LINE-1, Alu and Ribosomal DNA Repeats in Human Cell Lines Exposed to Radiofrequency Radiation
Source: Int J Mol Sci. 2023 May 27;24(11):9380. doi: 10.3390/ijms24119380 (PMC10253908; doi:10.3390/ijms24119380)
Supplement: Supplementary file 1 [file ijms-24-09380-s001.zip › Figure S1_CalibrationCurve.pdf]

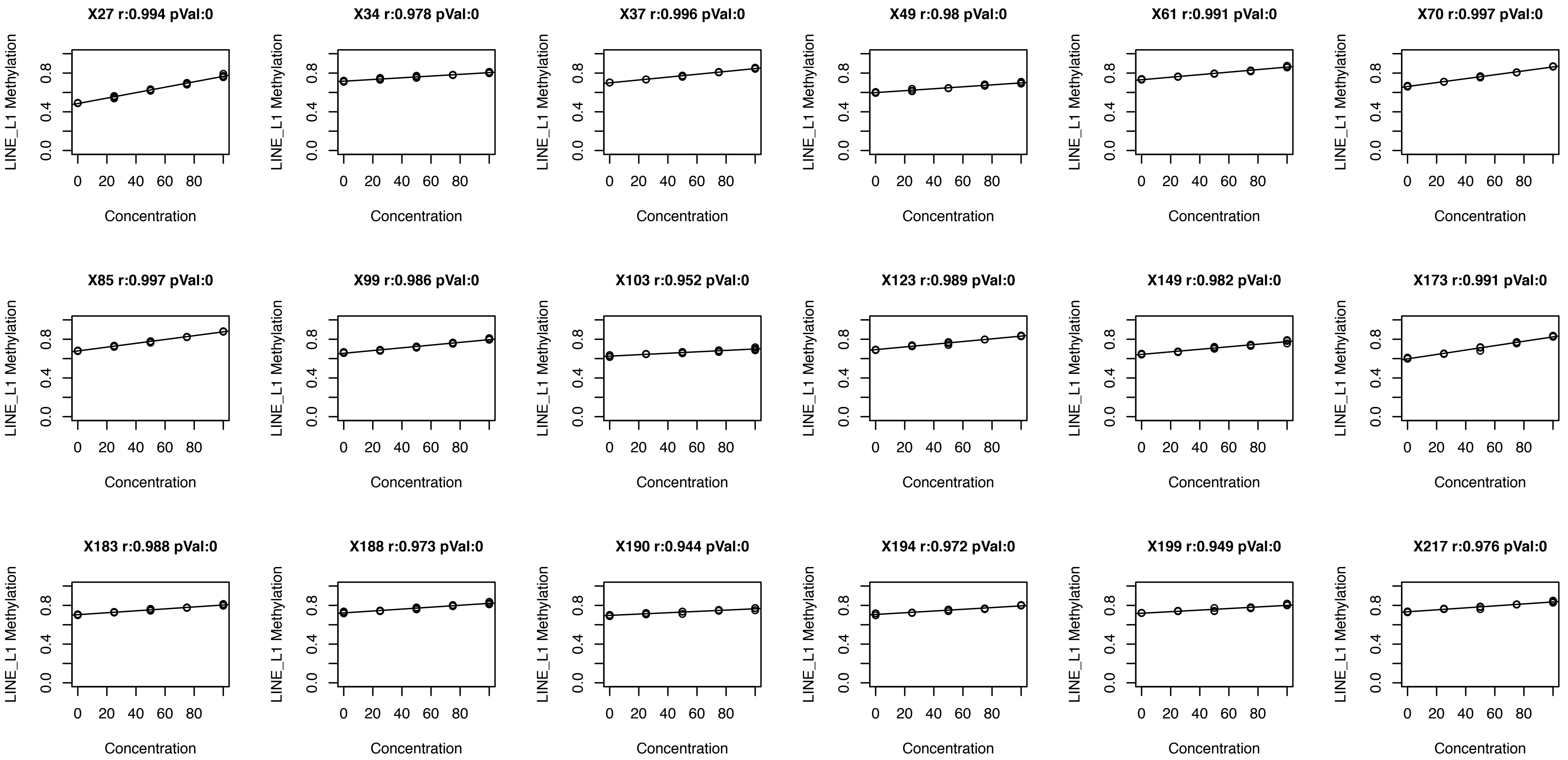
X27 r:0.994 pVal:0
X34 r:0.978 pVal:0
X37 r:0.996 pVal:0
X49 r:0.98 pVal:0
X61 r:0.991 pVal:0
X70 r:0.997 pVal:0
X85 r:0.997 pVal:0
X99 r:0.986 pVal:0
X103 r:0.952 pVal:0
X123 r:0.989 pVal:0
X149 r:0.982 pVal:0
X173 r:0.991 pVal:0
X183 r:0.988 pVal:0
X188 r:0.973 pVal:0
X190 r:0.944 pVal:0
X194 r:0.972 pVal:0
X199 r:0.949 pVal:0
X217 r:0.976 pVal:0
LINE_L1 Methylation
0.0
0.4
0.8
Concentration
0
20
40
60
80

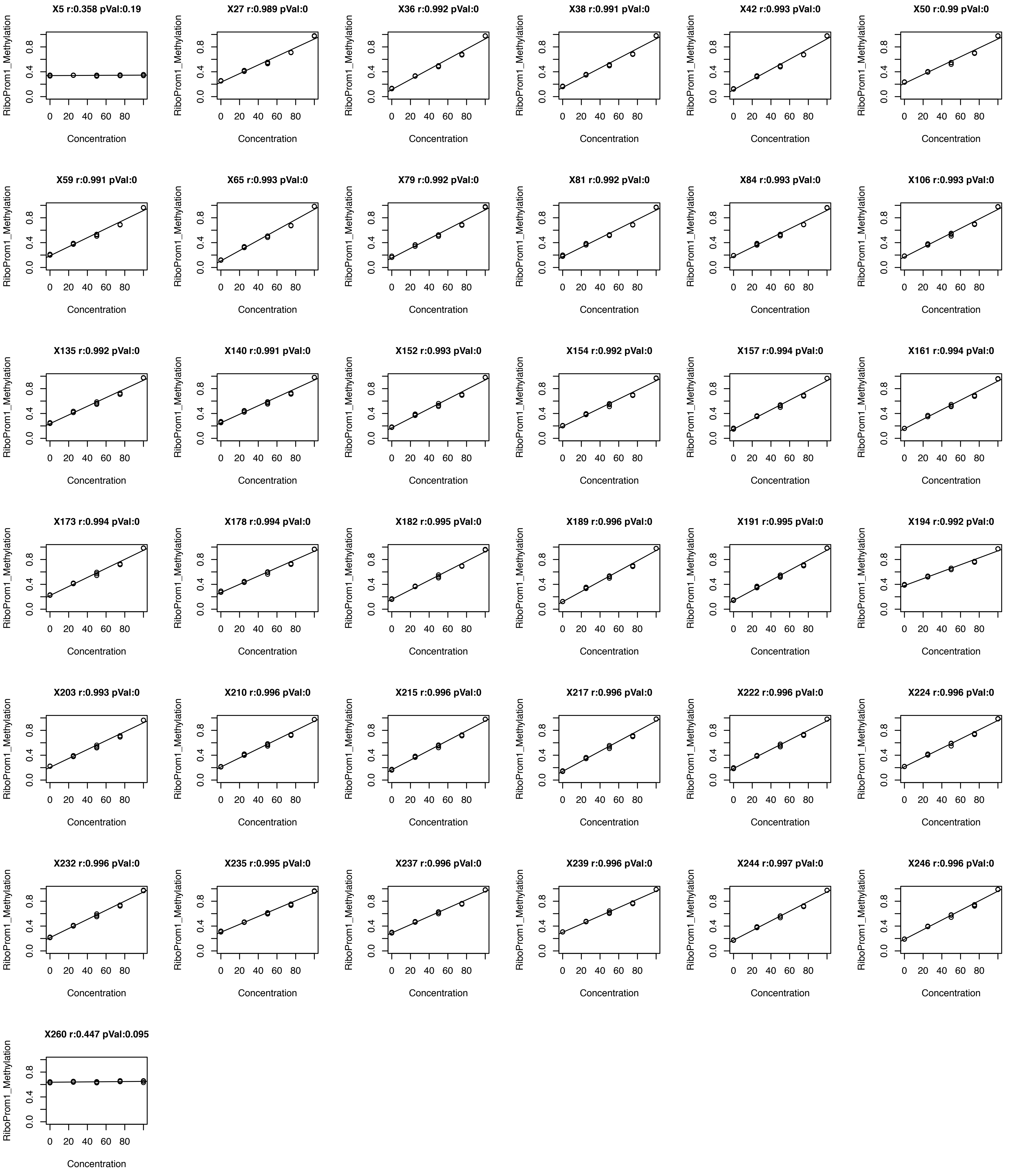
X5 r:0.358 pVal:0.19
X27 r:0.989 pVal:0
X36 r:0.992 pVal:0
X38 r:0.991 pVal:0
X42 r:0.993 pVal:0
X50 r:0.99 pVal:0
X59 r:0.991 pVal:0
X65 r:0.993 pVal:0
X79 r:0.992 pVal:0
X81 r:0.992 pVal:0
X84 r:0.993 pVal:0
X106 r:0.993 pVal:0
X135 r:0.992 pVal:0
X140 r:0.991 pVal:0
X152 r:0.993 pVal:0
X154 r:0.992 pVal:0
X157 r:0.994 pVal:0
X161 r:0.994 pVal:0
X173 r:0.994 pVal:0
X178 r:0.994 pVal:0
X182 r:0.995 pVal:0
X189 r:0.996 pVal:0
X191 r:0.995 pVal:0
X194 r:0.992 pVal:0
X203 r:0.993 pVal:0
X210 r:0.996 pVal:0
X215 r:0.996 pVal:0
X217 r:0.996 pVal:0
X222 r:0.996 pVal:0
X224 r:0.996 pVal:0
X232 r:0.996 pVal:0
X235 r:0.995 pVal:0
X237 r:0.996 pVal:0
X239 r:0.996 pVal:0
X244 r:0.997 pVal:0
X246 r:0.996 pVal:0
X260 r:0.447 pVal:0.095
RiboProm1_Methylation
0.0
0.4
0.8
0
20
40
60
80
Concentration

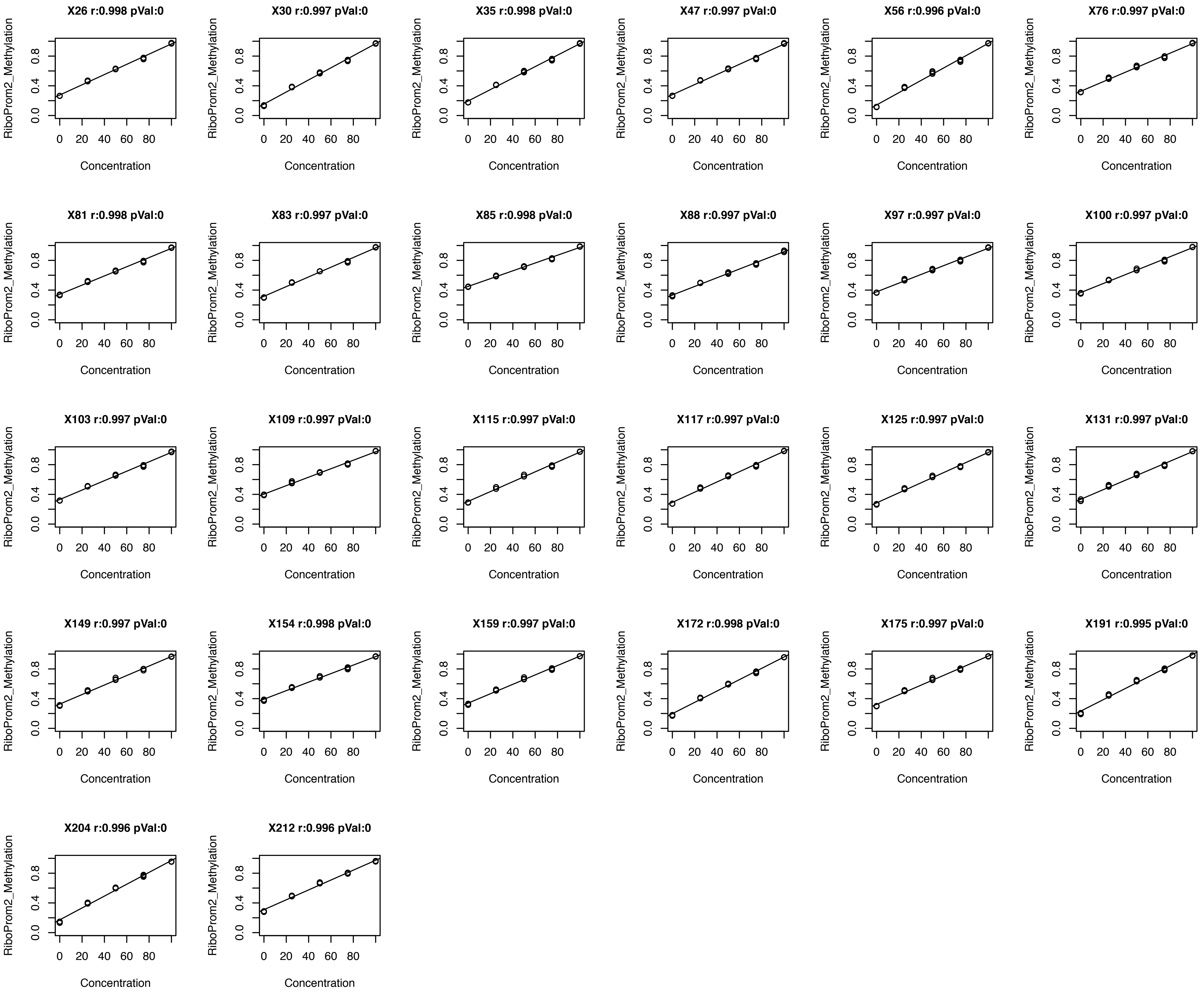
X26 r:0.998 pVal:0
X30 r:0.997 pVal:0
X35 r:0.998 pVal:0
X47 r:0.997 pVal:0
X56 r:0.996 pVal:0
X76 r:0.997 pVal:0
X81 r:0.998 pVal:0
X83 r:0.997 pVal:0
X85 r:0.998 pVal:0
X88 r:0.997 pVal:0
X97 r:0.997 pVal:0
X100 r:0.997 pVal:0
X103 r:0.997 pVal:0
X109 r:0.997 pVal:0
X115 r:0.997 pVal:0
X117 r:0.997 pVal:0
X125 r:0.997 pVal:0
X131 r:0.997 pVal:0
X149 r:0.997 pVal:0
X154 r:0.998 pVal:0
X159 r:0.997 pVal:0
X172 r:0.998 pVal:0
X175 r:0.997 pVal:0
X191 r:0.995 pVal:0
X204 r:0.996 pVal:0
X212 r:0.996 pVal:0
RiboProm2_Methylation
Concentration

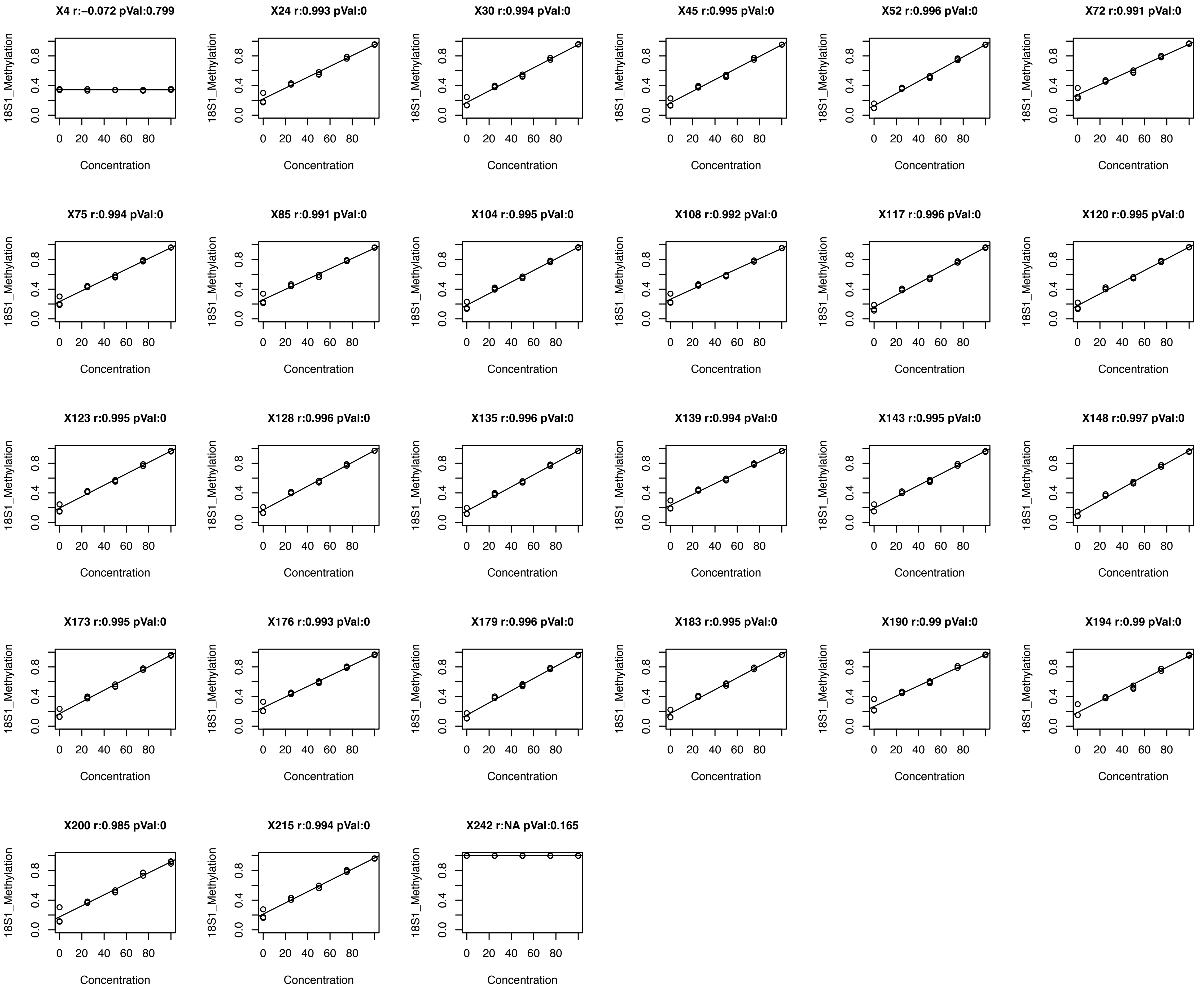
X4 r:−0.072 pVal:0.799
X24 r:0.993 pVal:0
X30 r:0.994 pVal:0
X45 r:0.995 pVal:0
X52 r:0.996 pVal:0
X72 r:0.991 pVal:0
X75 r:0.994 pVal:0
X85 r:0.991 pVal:0
X104 r:0.995 pVal:0
X108 r:0.992 pVal:0
X117 r:0.996 pVal:0
X120 r:0.995 pVal:0
X123 r:0.995 pVal:0
X128 r:0.996 pVal:0
X135 r:0.996 pVal:0
X139 r:0.994 pVal:0
X143 r:0.995 pVal:0
X148 r:0.997 pVal:0
X173 r:0.995 pVal:0
X176 r:0.993 pVal:0
X179 r:0.996 pVal:0
X183 r:0.995 pVal:0
X190 r:0.99 pVal:0
X194 r:0.99 pVal:0
X200 r:0.985 pVal:0
X215 r:0.994 pVal:0
X242 r:NA pVal:0.165
18S1_Methylation
0.0
0.4
0.8
Concentration
0
20
40
60
80

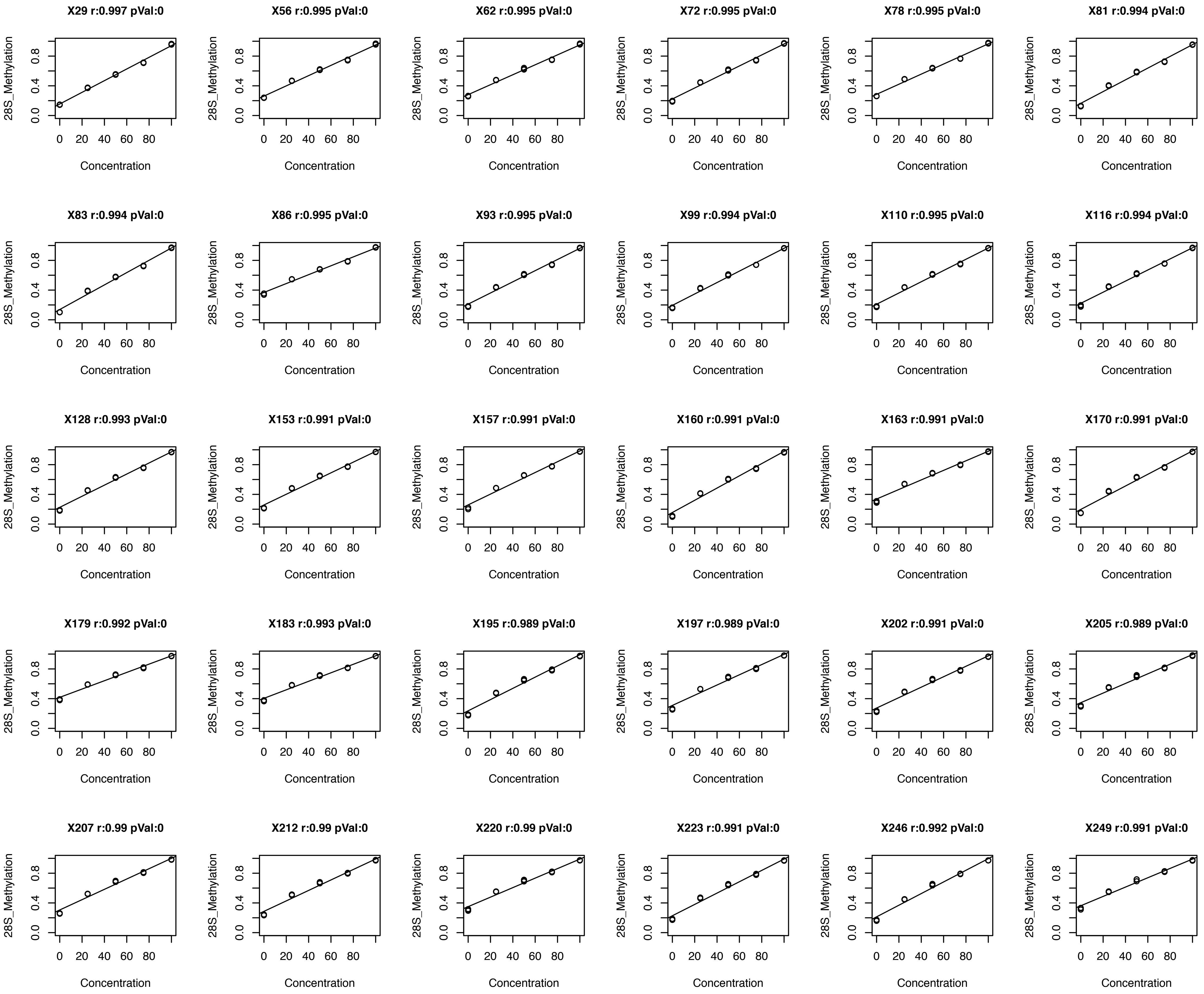
X29 r:0.997 pVal:0
X56 r:0.995 pVal:0
X62 r:0.995 pVal:0
X72 r:0.995 pVal:0
X78 r:0.995 pVal:0
X81 r:0.994 pVal:0
X83 r:0.994 pVal:0
X86 r:0.995 pVal:0
X93 r:0.995 pVal:0
X99 r:0.994 pVal:0
X110 r:0.995 pVal:0
X116 r:0.994 pVal:0
X128 r:0.993 pVal:0
X153 r:0.991 pVal:0
X157 r:0.991 pVal:0
X160 r:0.991 pVal:0
X163 r:0.991 pVal:0
X170 r:0.991 pVal:0
X179 r:0.992 pVal:0
X183 r:0.993 pVal:0
X195 r:0.989 pVal:0
X197 r:0.989 pVal:0
X202 r:0.991 pVal:0
X205 r:0.989 pVal:0
X207 r:0.99 pVal:0
X212 r:0.99 pVal:0
X220 r:0.99 pVal:0
X223 r:0.991 pVal:0
X246 r:0.992 pVal:0
X249 r:0.991 pVal:0
28S_Methylation
Concentration
0.0
0.4
0.8
0
20
40
60
80

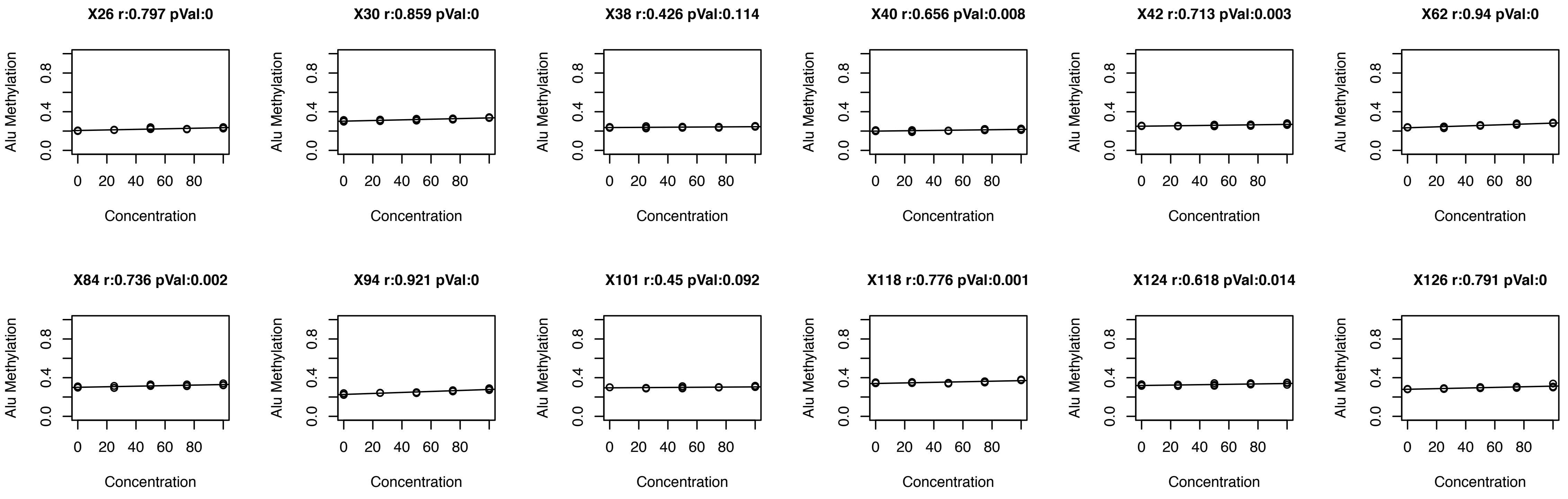

Figure S1. DNAm calibration curves for rDNA, LINE1 and Alu targets. Plots reporting DNAm profiles for rDNA, LINE1 and Alu amplicons assessed using calibrator samples at increasing DNAm percentages (0%, 25%, 50%, 75%, 100%). Calibration curves are obtained through linear regression between measured DNAm levels and expected ones. r-value = Spearman correlation coefficient; p-value = linear regression statistical significance coefficient.
