# Supplementary material for: Evaluation of DNA Methylation Profiles of LINE-1, Alu and Ribosomal DNA Repeats in Human Cell Lines Exposed to Radiofrequency Radiation
Source: Int J Mol Sci. 2023 May 27;24(11):9380. doi: 10.3390/ijms24119380 (PMC10253908; doi:10.3390/ijms24119380)
Supplement: Supplementary file 1 [file ijms-24-09380-s001.zip › Figure S2_Amplicon_Sequence.pdf]

Figure S2. Genomic sequences of rDNA, LINE-1 and Alu amplicons. List of genomic sequences amplified by rDNA, LINE-1 and Alu amplicons assayed through target-bisulfite sequencing. CpGs are highlighted and identified via their relative position within each amplicon.

>18S1

4 24 30 45 52
TAGCCGTTTCTCAGGCTCCCTCTCCGGAATCGAACCCTGATTCCCCGTCACCCGTGGTCACCATGGTAGGCA
72 75 85 104 108 117 120 123 128 135 139
CGGCGACTACCATCGAAAGTTGATAGGGCAGACGTTCGAATGGGTCGTCGCCGCCACGGGGGGCGTGCGA
143 148 173 176 179 183 190 194 200
TCGGCCCGAGGTTATCTAGAGTCACCAAAGCCGCCGGCGCCCGCCCCCGGCCGGGGCCGGAGAGGGGCT
215 242
GACCGGGTTGGTTTTGATCTGATAAATGCACG

>28S

29 56 62
GTATTTAGCCTTAGATGGAGTTTACCACCCGCTTTGGGCTGCATTCCCAAGCAACCCGACTCCGGGAAGACC
72 78 81 83 86 93 99 110 116 128
CGGGCCCGGCGCGCCGGGGGCCGCTACCGGCCTCACACCGTCCACGGGCTGGGCCTCGATCAGAAGGACT
153 157 160 163 170 179 183 195 197 202 205 207
TGGGCCCCCACGAGCGGCGCCGGGGAGCGGGTCTTCCGTACGCCACATGTCCCGCGCCCCGCCGCGGGG
212 220 223 246 249
CGGGGATTCGGCGCTGGGCTCTTCCCTGTTCACTCGCCGTTACTGAGGGAATCCTGGTTAGTT

>Alu

26 30 38 40 42 62
TCTCTACTAAAAATACAAAAATTAGCCGGGCGTGGTGGCGCGCCTGTAATCCCAGCTACTCGGGAGGCT
84 94 101 118 124 126
GAGGCAGGAGAATCGCTTGAACCCGGGAGGCGGAGGTTGCAGTGAGCCGAGATCGCGCCACTGCACTCC

AGCCTGG

>LINE-L1

27. 34. 37 49 61 70
CTCTGAGCCAGGTGTGGGATATAGTCTCGTGGTGCGCCGTTTCTTAAGCCGGTCTGAAAAGCGCAATATTC
85 99 103 123
GGGTGGGAGTGACCCGATTTTCCAGGTGCGTCCGTCACCCCTTTCTTTGACTCGGAAAGGGAACTCCCTGA
149 173 183 188 190 194 199
CCCCTTGCGCTTCCCAGGTGAGGCAATGCCTCGCCCTGCTTCGGCTCGCGCACGGTGCGCACACACACTGG
217
CCTGCGCCCACTGTCTGGCACTCCCTAGTGAG

>RiboProm1

5 27 36 38 42 50 59 65
GTCCCCGCTGTGAGCTAGGCAGAGCTCCGGAAAGCCCGCGGTCGTCAGCCCGGCTGGCCCGGTGGCGCCA
79 81 84 106 135
GAGCTGTGGCGCGTCGCTTGTGAGTCACAGCTCTGGCGTGCAGGTTTATGTGGGGGAGAGGCTGTCGCTG
140 152 154 157 161 173 178 182 189 191 194 203
CGCTTCTGGGCCCGCGGCGGGCGTGGGGCTGCCCGGGCCGGTCGACCAGCGCGCCGTAGCTCCCGAGGCC
210 215 217 222 224 232 232 235 237 244 246 260
CGAGCCGCGACCCGCGGGGACCCGCCGCGCGTGGCGCGGGAGGCTGGGACGCCCTTCC

>RiboProm2

26 30 35 47 56

GTGTCCTTGGGTTGACCAGAGGGCCCCGGGCGCTCCGTGTGTGGCTGCGATGGTGGCGTTTTTGGGGACA

76 81 83 85 88 97 100 103 109 115 117 125 131

GGTGTCCGTGTCGCGCGTCGCCTGGGCCGGCGGCGTGGTCGGTGACGCGACCTCCCGGCCCCGGGGGAG

149 154 159 172 175 191 204

GTATATCTTTCGCTCCGAGTCGGCATTTTGGGCCGCCGGGTTATTGCTGACACGCTGTCCTCTGGCGACCTG

212

TCGCTGGAGAGGTTGGGCCTTGGGATG
